# Biochemical and cellular consequences of the antithrombin p.Met1? mutation identified in a severe thrombophilic family

**DOI:** 10.18632/oncotarget.26059

**Published:** 2018-09-04

**Authors:** José Navarro-Fernández, María Eugenia de la Morena-Barrio, Emma Martínez-Alonso, Ingunn Dybedal, Mara Toderici, Nataliya Bohdan, Antonia Miñano, Ketil Heimdal, Ulrich Abildgaard, José Ángel Martínez-Menárguez, Javier Corral, Vicente Vicente

**Affiliations:** ^1^ Servicio de Hematología y Oncología Médica, Hospital Universitario Morales Meseguer, Centro Regional de Hemodonación, Universidad de Murcia, IMIB-Arrixaca, CIBERER, Murcia, Spain; ^2^ Department of Cellular Biology and Histology, Faculty of Medicine, University of Murcia, Murcia, Spain; ^3^ Department of Haematology, Oslo University Hospital, Oslo, Norway; ^4^ Department of Medical Genetics, Oslo University Hospital, Oslo, Norway

**Keywords:** antithrombin, thrombosis, initiation codon, translation

## Abstract

Nature is always the best inspiration for basic research. A family with severe thrombosis and antithrombin deficiency, the strongest anticoagulant, carried a new mutation affecting the translation-start codon of *SERPINC1*, the gene encoding antithrombin. Expression of this variant in a eukaryotic cell system produced three different antithrombins. Two downstream methionines were used as alternative initiation codons, generating highly expressed small aglycosylated antithrombins with cytoplasmic localization. Wild-type antithrombin was generated by the use of the mutated AUU as initiation codon. Actually, any codon except for the three stop codons might be used to initiate translation in this strong Kozak context.

We show unexpected consequences of natural mutations affecting translation-start codons. Downstream alternative initiation AUG codons may be used when the start codon is mutated, generating smaller molecules with potential different cell localization, biochemical features and unexplored consequences. Additionally, our data further support the use of other codons apart from AUG for initiation of translation in eukaryotes.

## INTRODUCTION

It is well accepted that in most eukaryotic mRNAs the selection of the translational initiation site occur via a scanning mechanism which identify a start AUG codon. Thus, mutations disturbing this initiation codon will probably have important biological consequences. Indeed, there are examples of diseases caused by mutations affecting initiation AUG codons [1]. However, most of studies identifying mutations affecting this codon in patients are restricted to the genetic characterization of the mutation and to family studies to demonstrate the cosegregation with the disease phenotype. Actually, current mutation databases, described incorrectly the missense consequence extrapolated from the effect of the mutation on the affected codon, when that amino acid change probably will never happen. For example, in the Human Gene Mutation Database (HGMD), the five mutations affecting the initiation codon of *F8*, the gene encoding FVIII, identified in patients with haemophilia A, all are considered as missense mutations (Met-Thr; Met-Arg; Met-Ile; Met-Val and Met-Leu). The identification and the characterization of new natural mutations affecting initiation codons may reveal both new information on this key biological process and the pathogenic consequences of disturbing a correct initiation of translation.

Antithrombin is a serpin that inhibits multiple procoagulant serine proteases, mainly thrombin and factor Xa, by an efficient mechanism. Consequently, antithrombin plays a key anticoagulant role by correctly controlling and preventing inappropriate, excessive or mislocalized clotting of blood, which may cause venous thromboembolism (VTE). [2] Thus, antithrombin deficiency was the first and so far strongest thrombophilic factor, identified 50 years ago, with autosomic and dominant features. [3] Accordingly, antithrombin deficiency is evaluated in patients with early or recurrent thrombosis, as well as in cases with familial history of thrombosis or with thrombosis at unusual localizations [4]. Analysis of antithrombin and *SERPINC1*, the gene encoding this anticoagulant, in patients with deficiency has been very helpful to identify mechanisms involved in its deficiency, as well as to describe key functional residues of this anticoagulant. The best example is the identification of residues involved in the interaction with heparin, a cofactor that fully activates the anticoagulant capacity of antithrombin [5]. Moreover, these studies may also assist to identify prognostic markers, as there is a considerable clinical heterogeneity among patients with reduced antithrombin activity [6]. When the mutation impairs the transcription, the RNA stability, the folding or the secretion of the molecule, no variant antithrombin is detected in plasma. This quantitative defect is named type I deficiency, is rare in the general population, and significantly increases the risk of VTE. Alternatively, when the mutation does not significantly impair the secretion of the variant molecule but reduces or abolish its anticoagulant activity causes a qualitative deficiency that is called type II deficiency. Type II deficiencies are more frequent in the general population and usually associate with milder risk of VTE [7], although for certain mutations, the variant associates with stronger clinical severity than mutations causing type I deficiency, because the mutation may also have a dominant negative effect [8]. The characterization of families with antithrombin deficiency and very severe clinical phenotype might assist to identify new mechanisms associated with the risk of VTE.

Antithrombin is a glycoprotein synthesized by hepatocytes as a 464 amino acid precursor. As for most of secretory proteins, antithrombin contains a signal peptide (32 amino acids for antithrombin), which is crucial for introducing the nascent protein into the endoplasmic reticulum (ER). Translocation of the protein into the ER is followed by post-translational processing, which includes disulphide bond formation, N-glycosylation, signal peptide cleavage, and strict protein folding into a metastable conformation, which is required for its efficient mechanism of inhibition of this serpin [9].

The present work has characterized a new *SERPINC1* mutation identified in a family with antithrombin deficiency that displayed very severe clinical phenotype. The study revealed surprising results affecting classical concepts of cellular and molecular biology that open new perspectives on the pathological relevance of mutations disturbing the initiation codon.

## RESULTS

### Clinical data

**Figure 1:**
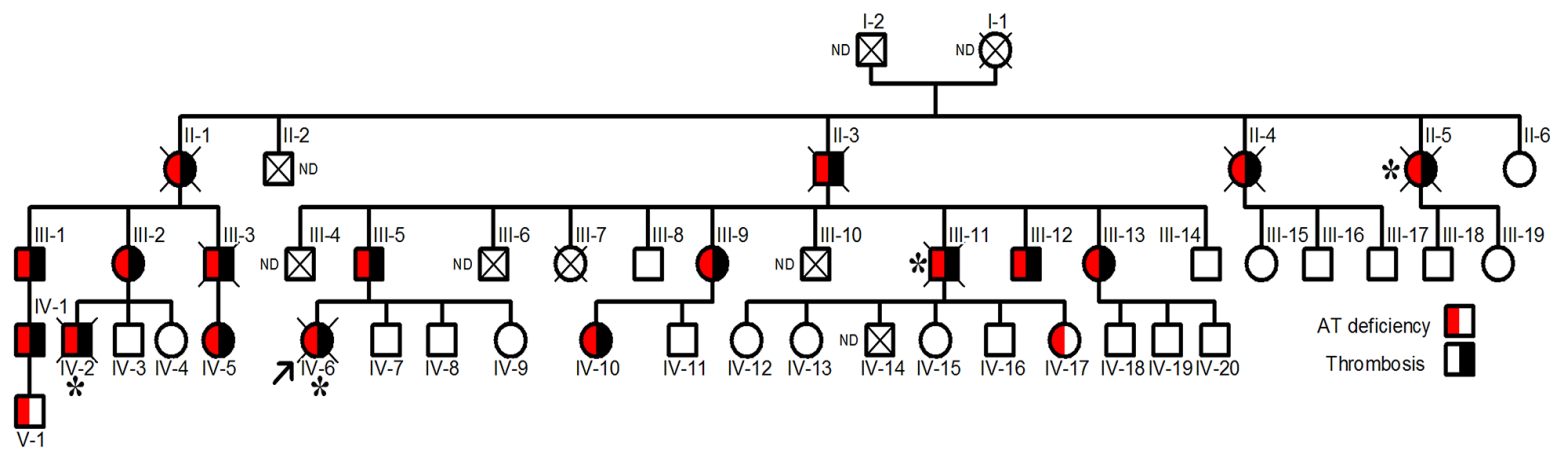
Pedigree of the Norwegian thrombophilic family The presence of thrombotic events and/or antithrombin deficiency is indicated by symbols filled in black and red, respectively. The arrow points the proband. Carriers who died from thromboembolic events are indicated by ^*^. ND: Not determined.

The proband, a Norwegian woman, was diagnosed of renal vein thrombosis at the age of 14. Multiple recurrences appeared during her short life, as a massive cerebral thrombotic occlusion occurring at the age of 26 caused her death. Thrombophilic analysis was done in the proband. Levels of protein C and protein S were found repeatedly normal at occasions when she was only treated with low molecular weight heparin. The proband did not carry prothrombotic polymorphisms, prothrombin G2010A and factor V Leiden. Lupus anticoagulant was not present. The JAK-2 V617F mutation was not detected. The single thrombophilic defect identified was antithrombin deficiency, with anti-FXa values of 55% and antigen levels of 56% compared with that observed in a pool of 100 healthy blood donors (100%). These results suggest a type I deficiency. Eighteen relatives had antithrombin deficiency with a mean anti-FXa activity of 55±2.6% and antigen levels of 56±1.8%, and all except two young carriers, developed early and/or recurrent venous or arterial thrombotic events, in four cases with fatal consequences (Figure 1).

### Genetic study

Sequence of *SERPINC1* in the proband revealed a c.3G>T heterozygous mutation in exon 1 that affects the translation-initiation codon (Supplementary Figure 1). This mutation predicts a missense change p.(Met1Ile), but it theoretically abolished protein translation, which might explain the type I deficiency of carriers. This mutation has not been previously described (http://www.hgmd.cf.ac.uk/ac/all.php). However, other mutation affecting the same AUG codon with different consequence p.(Met1Thr) has been identified in a Japanese patient [10]. Interestingly, the patient and her father carrying this different mutation affecting the initiation AUG codon also had early venous and arterial thrombotic events [10].

Sequencing of *SERPINC1* in 4 relatives with antithrombin deficiency and relevant thrombotic history, including pulmonary embolism, stroke and recurrent thrombosis, confirmed the presence of the c.3G>T mutation in heterozygous state in all patients.

### Biochemical characterization of antithrombin deficiency

Electrophoretic and Western blot analysis of plasma antithrombin confirmed the reduction of levels among carriers of the *SERPINC1* c.3G>T mutation (Supplementary Figure 2). No abnormal forms of antithrombin were identified in plasma of carriers (Supplementary Figure 2). All these data confirmed a type I antithrombin deficiency. Similarly Japanese carriers of p.(Met1Thr) mutation also had type I antithrombin deficiency [10].

### Recombinant model

The extraordinary severe clinical phenotype associated with this mutation, which is even more severe than that found in patients with nonsense mutations leading to type I deficiency from our cohort of 250 unrelated patients with congenital antithrombin deficiency, encouraged us to perform further studies. *in vitro* studies were designed to test if this mutation might induce the expression of an aberrant antithrombin with a potential gain of function that might help to explain the clinical phenotype of carriers. Accordingly, recombinant expression of this mutation was done in HEK-EBNA eukaryotic cells.

**Figure 2:**
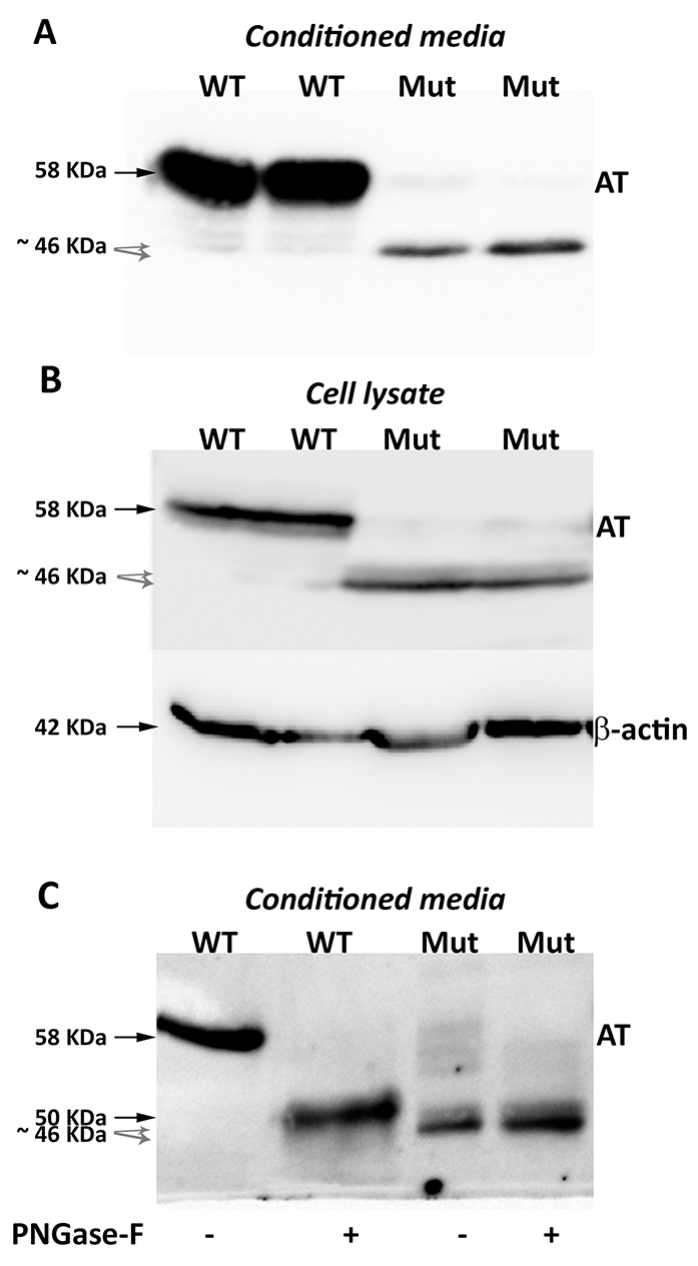
Antithrombins expressed by HEK-EBNA cells 24 h after transfection with pCEP4-S169A (WT) and pCEP4-S169A-M1I (Mut) plasmids Proteins were identified by SDS-PAGE and western blot using an anti-human antithrombin polyclonal antibody. **(A)** Antithrombin (AT) released to the conditioned media. The same volume of conditioned was used for cells transfected with both plasmids. **(B)** Intracellular antithrombin and beta-actin expression. The same amount of cell lysates was used for cells transfected with both plasmids. **(C)** Glycomic analysis of recombinant antithrombins purified from the conditioned media. The wild-type protein and the small antithrombins were treated (+) or not (-) with N-glycosidase F (PNGase-F) and detected by western blot. Black arrows point the wild-type antithrombin. Grey dashed arrows indicate the small variants. Estimated molecular weights are also indicated.

Surprisingly, Western blot analysis with the polyclonal antibody against human antithrombin of conditioned media of HEK-EBNA cells transfected with the mutated plasmid revealed three proteins, although expression was 5-20-fold lower than that observed in cells transfected with the wild-type plasmid (Figure 2). One band had the same electrophoretic mobility as the wild-type antithrombin (58 KDa) while two smaller bands were also detected (Figure 2A). These small forms (∼46 KDa) although had lower expression than the wild-type molecule, still showed a high intracellular expression (Figure 2B).

Purification of proteins from the conditioned media was done following the same strategy to purify plasma antithrombin: heparin affinity and ion exchange, supporting that all recombinant proteins bind heparin.

Proteomic analysis of recombinant antithrombins confirmed that all proteins corresponded to human antithrombin. For the small molecules, 19 peptides, covering 57% of the antithrombin sequence, were identified (Supplementary Figure 3). The ATEDEGSEQK (residues 26-35) was the first aminoterminal peptide identified in the smaller proteins, but the first aminoterminal peptides of the wild-type antithrombin: HGSPVDICTAKPR and DIPMNPMNPMCIYR were not detected in the small antithrombins (Supplementary Figure 3). Neither mutations nor aberrant post-translational modifications were detected by proteomic analysis.

Treatment of conditioned media with PNGase-F confirmed that the 58 KDa band corresponded to the fully glycosylated wild-type antithrombin (Figure 2C). Interestingly, the 46 KDa bands did not change their electrophoretic mobility after treatment with PNGase-F and they were still smaller than aglycosylated wild-type antithrombin (Figure 2C), supporting that these aberrant and smaller antithrombins had no N-glycosylation but also have lost few residues. Unfortunately, Edman’s sequencing of these smaller antithrombins rendered no results because of insufficient purified protein.

**Figure 3:**
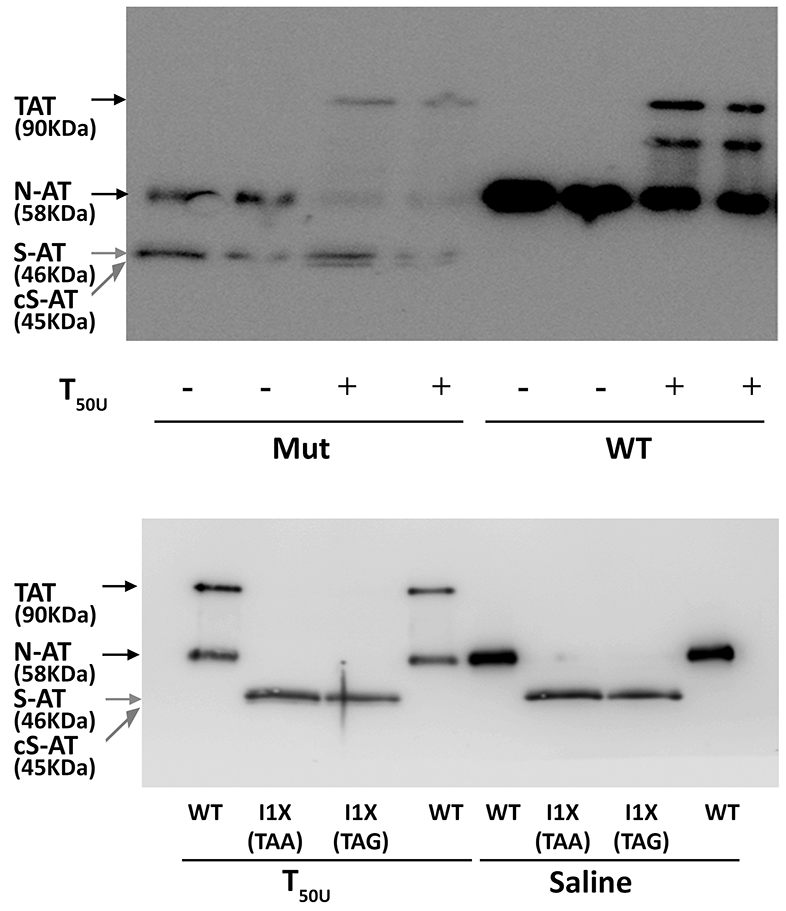
Anti-thrombin activity of antithrombin from conditioned media of HEK-EBNA cells transfected with wild-type (WT), the pCEP4-S169A-M1I (Mut), pCEP4-S169A-I1X (TAA) and pCEP4-S169A-I1X (TAG) plasmids The medium was incubated with (+) or without (-) 50 units of thrombin (T_50U_). Electrophoretic separation with SDS-PAGE under reducing conditions was followed by immunodetection of antithrombins with an anti-human antithrombin polyclonal antibody. Arrows point native wild-type antithrombin (58 KDa) (N-AT), Thrombin-antithrombin complexes (TAT) (90 KDa), small antithrombins (46 KDa) (S-AT), and cleaved small antithrombins (45 KDa) (cS-AT). Five-fold conditioned media of mutants was loaded compared to the wild-type.

Functional studies confirmed that the 58 KDa protein also had anticoagulant activity, as covalent 90 KDa thrombin-antithrombin complexes were formed (Figure 3). In contrast, the small 46 KDa proteins did not form complexes with thrombin but seemed to behave as a substrate for this protease, as a smaller band (45 KDa) was detected when incubated with thrombin (Figure 3). Functional studies done with conditioned media of cells transfected with plasmids containing a stop codon at position 1, which produce small 46 KDa antithrombins but did not produce wild-type antithrombin (see later), confirmed that the 46 KDa proteins did not form thrombin-antithrombin complexes (Figure 3) and had no anti-FXa activity in a chromogenic assay (Supplementary Figure 4).

**Figure 4:**
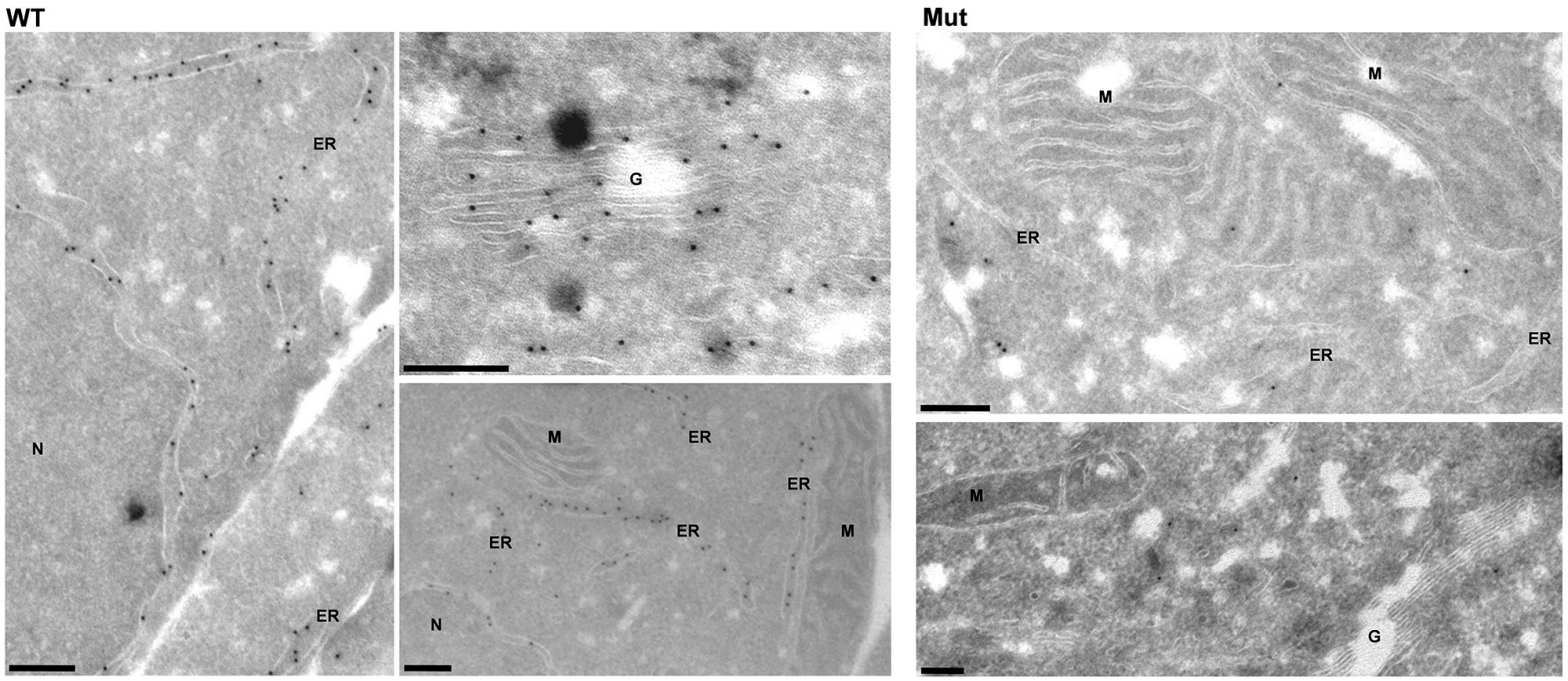
Intracellular expression pattern of antithrombin in HEK-EBNA cells 24 h after transfection with wild-type (WT) and mutant plasmids (Mut) Wild-type antithrombin is specifically located in the endoplasmic reticulum (ER) and the Golgi complex (G) whereas the mutant is not present in these compartments, showing a cytosolic pattern ER: Endoplasmic reticulum, N: Nucleus, G: Golgi complex, M: Mitochondria. Bars, 200 nm.

Analysis of intracellular expression of antithrombin by immunofluorescence showed a diffuse pattern in cells transfected with the plasmid containing the c.3G>T mutation compared to the expression observed in cells transfected with the wild-type plasmid (Supplementary Figure 5). Interestingly, immunoelectron-microscopy revealed significant differences in the intracellular distribution of antithrombin. HEK-EBNA cells transfected with the wild-type plasmid had a strong and specific expression of antithrombin in the ER and Golgi apparatus (Figure 4). In contrast, cells transfected with the plasmid carrying the c.3G>T mutation showed a diffuse and mild expression of antithrombin with cytoplasmic localization, but no significant signals neither in the ER nor the Golgi complex (Figure 4), suggesting that mutation impaired antithrombin enter into the secretory pathway.

### Site directed mutagenesis to verify the use of alternative initiation codons

Site directed mutagenesis of the pCEP4-S169A-M1I plasmid were designed to evaluate whether alternative translation-initiation codons might be used in the recombinant model to produce the three antithrombins detected by Western blot (Supplementary Table 1).

**Figure 5:**
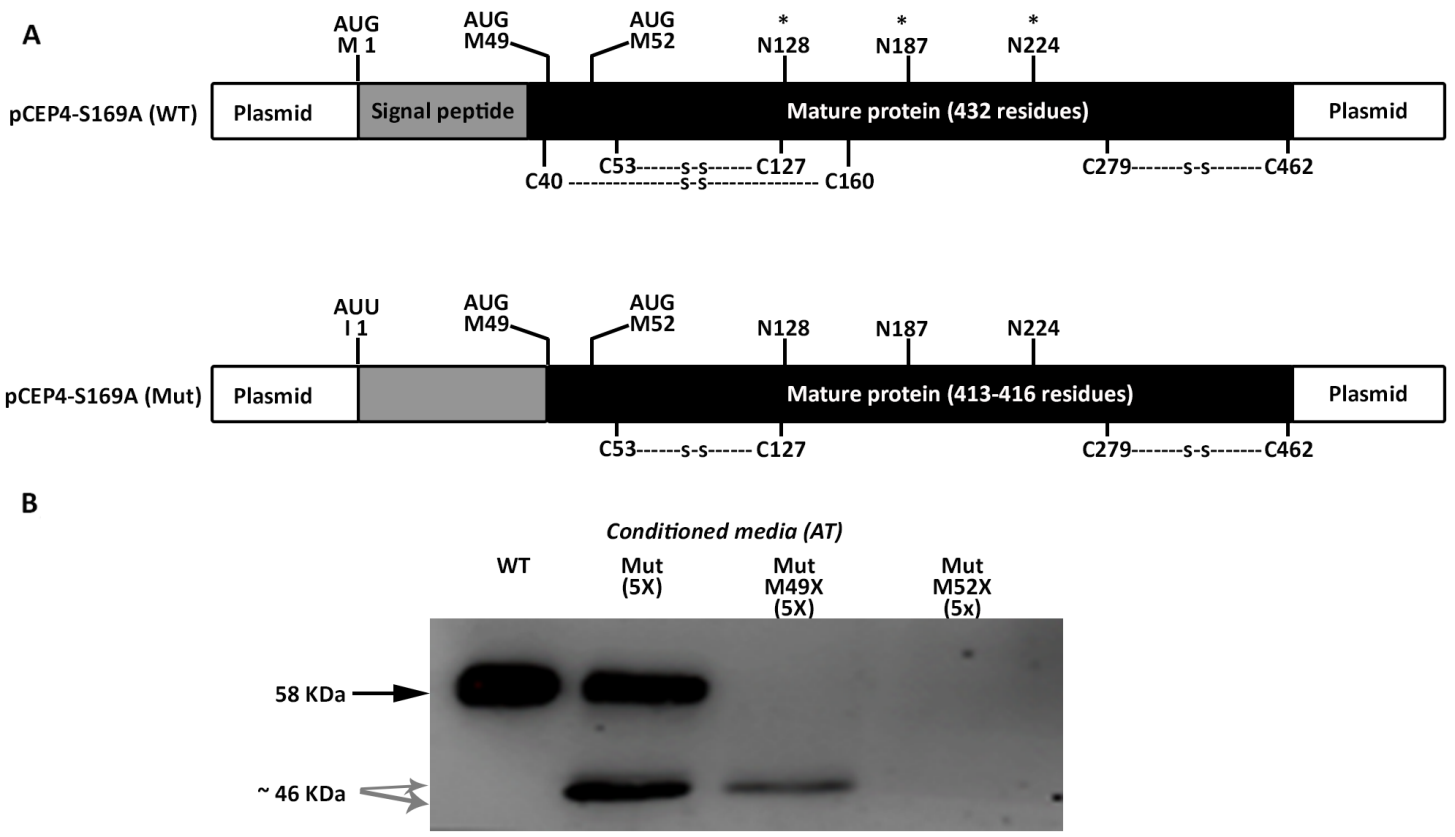
Identification of alternative AUG initiation codons in *SERPINC1* responsible for the small antithrombins expressed in cells transfected with pCEP4-S169A-M1I (Mut) plasmid **(A)** Squematic representation of AUG codons identified in the pCEP4-S169A (WT) and pCEP4-S169A-M1I (Mut) plasmids used for transfection. Potential initiation codons, localization of the signal peptide, and the mature proteins with potential post-translational modifications are shown. S: disulfide linked bonds; ^*^ N-glycosylation. **(B)** Antithrombin (AT) released to conditioned media of cells transfected with WT and Mut plasmids, as well as with mutated pCEP4-S169A-M1I plasmids that also contained nonsense mutations for Met49 and Met52. Antithrombins were immunodetected after Western blot. Full length wild-type antithrombin (58 KDa) is pointed by an arrow while small antithrombins (46 KDa) are pointed by grey arrows. Five-fold conditioned media of mutants was loaded compared to the wild-type.

We firstly tried to clarify the small antithrombins, which according to the electrophoretic, glycomic, proteomic and electron microscopy data, might be explained by the use of down-stream in frame methionines. In human antithrombin the first two in frame methionines excluding the initiation codon are located at positions Met49 and Met52; 17 and 20 residues behind the signal peptide, in the mature protein (Figure 5A). Mutation of the codons encoding Met49 and Met52 to stop codons, confirmed that these methionines were used as alternative translation-initiation codons when the initiation codon encoding methionine 1 (Met1) was mutated (Figure 5B). The context of these methionines has a high score for potential initiation of translation (0.670 and 0.466, respectively vs. 0.938 for the wild-type Kozak sequence surrounding Met1 in *SERPINC1* mRNA and 0.843 for the Kozak sequence surrounding Met1 in the plasmid used for recombinant expression) [11].

**Figure 6:**
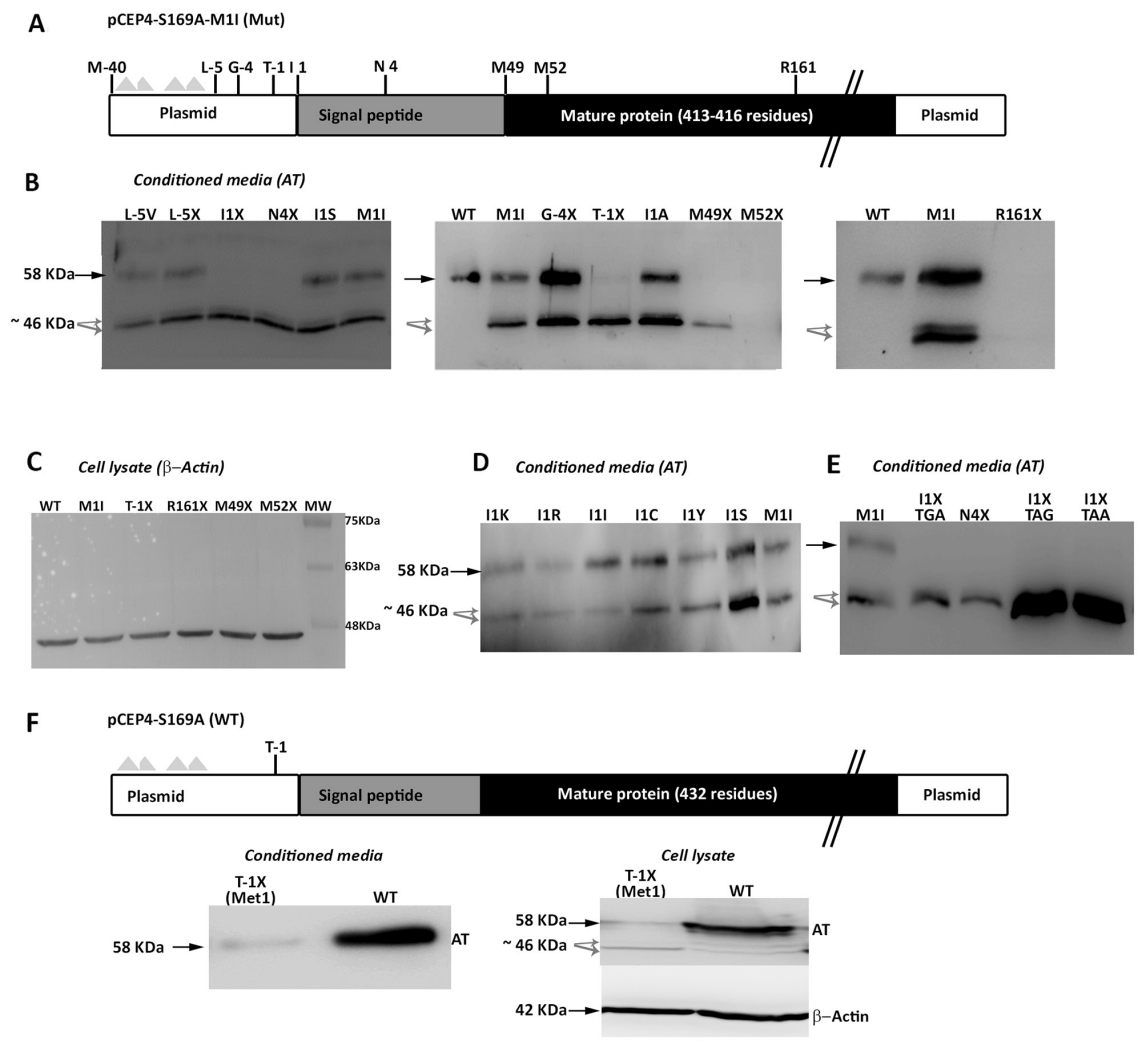
Identification of non AUG initiation codons in *SERPINC1* **(A)** Squematic representation of the mutated pCEP4-S169A-M1I plasmid used in the recombinant model showing residues mutated to identify alternative initiation codons. Triangles represent stop codons in the reading frame of *SERPINC1*. **(B)** Antithrombin released to conditioned media (AT) of cells transfected with the pCEP4-S169A-M1I plasmid containing different mutations: missense or nonsense mutations affecting residues located before the original initiation codon (L-5V, L-5X; G-4X; T-1X); missense or nonsense mutations at or just after the original initiation codon (I1S, I1X, N4X); nonsense mutation affecting the first downstream AUG codons (M49X, M52X); and a nonsense mutation downstream of all potential initiation codons (R161X). **(C)** Intracellular expression of beta-actin as loading control for selected mutants, including those with no antithrombin released to the conditioned media (M52X and R161X). **(D)** Consequence on antithrombin from conditioned media of mutations affecting Ile1 in the pCEP4-S169A-M1I plasmid. **(E)** Effect on antithrombin from conditioned media of possible nonsense mutations affecting position 1 in the pCEP4-S169A-M1I plasmid. **(F)** Squematic representation of the wild-type pCEP4-S169A plasmid showing the mutation that severely disturbs the Kozak sequence (T-1X) in a wild-type context (Met1). Antithrombin from conditioned media and cell lysate as well as intracellular beta-actin were detected by Western blot. Full length wild-type antithrombin (58 KDa) is pointed by an arrow while small antithrombins (46 KDa) are pointed by grey arrows. Five-fold conditioned media of mutants was loaded compared to the wild-type.

We initially proposed two hypotheses to explain the presence of a wild-type molecule in cells transfected with the c.3G>T mutation: contamination with wild-type plasmid or an alternative initiation methionine up-stream, in the plasmid sequence. We identified an AUG codon keeping the reading frame of antithrombin, located 40 residues up-stream (Figure 6). However, mutation of this residue to an isoleucine codon, did not modify the expression pattern, and the three antithrombins were still detected in the conditioned media (data not shown). Indeed, there are 4 stop codons downstream this -40 AUG codon (Figure 6A). This experiment, together with the nonsense mutagenesis disturbing Met49 and Met52 (M49X and M52X, respectively), which did not produced the wild-type antithrombin (Figure 5) and the mutation of arginine 161 to a stop codon (R161X) that produced no antithrombin at all (Figure 6B), discarded any potential contamination with wild-type plasmid. Thus, we explored the potential use of other codon, different than the AUG, as initiation of translation to explain the wild-type antithrombin detected in our eukaryotic cell model when transfected with the mutant plasmid. The first candidate was a CUG codon (coding for leucine), located 5 positions upstream, as this codon may initiate translation in eukaryotes [12]. However, neither mutation to valine (L-5V) nor to stop (L-5X), abolished the expression of the wild-type antithrombin (Figure 6). To localize the new translation-initiation site, codons surrounding Ile1 were mutated to stop codons. While a stop codon in Thr-1 (T-1X) did still produce the three antithrombins, a stop codon in Asn4 (N4X) only rendered the two small antithrombins (Figure 6C). These data, together with the high score for initiation of translation of codon 1 in the plasmid (0.843) strongly suggest that the AUU codon encoding isoleucine might be used as initiation of translation. Ten site directed mutagenesis were done in the AUU codon encoding Ile1 to test different nucleotides in each position of this initiation codon. All tested combinations produced the three antithrombins at different levels (Figure 6D), except those encoding stop codons, which only produced the two small antithrombins (Figure 6E).

Finally, we also disturbed the Kozak sequence in the wild-type plasmid (M1), maintaining the initiation AUG codon. A stop codon at Thr-1 (T-1X) also caused a severe reduction of wild-type antithrombin in the conditioned media, but interestingly, also rendered the intracellular expression of small antithrombin (Figure 6F).

## DISCUSSION

There are few studies evaluating the consequences of mutations disturbing translation initiation codons in recombinant models, as it is generally considered these mutations cause the loss of the affected molecule by abolishing translation. In some cases the mutation lead to loss of function, finding no expression of the variant [13, 14], but few studies showed the use of in-frame downstream methionines [15, 16]. Nevertheless, translation may not necessarily occur from the first downstream alternative start site, or from a single alternative start site [15, 16]. An interesting example is a second-site mutation in the initiation codon of *WAS* (c.1A>T) that allows the use of a close downstream AUG codon (p.M1-P5del). This alternative initiation bypass the pathogenic consequences of the c.11delG; p.G4fsX40 [17].

There are some gene variations affecting this codon or the surrounding Kozak sequence with mild consequences according to the high frequency of the mutated allele. One excellent example is the rs291466 SNP affecting the initiation AUG codon of the 3-hydroxyisobutyryl-CoA hydrolase (HIBCH). The c.2T>C mutation causes a missense change of the initiation methionine codon to threonine. Interestingly, the minor-allele frequency of this SNP is very high (0.43), and homozygous carriers of this SNP have higher methylmalonic acid expression than carriers of the wild-type allele encoding for methionine at position 1 [18].

Thus, the study of new natural mutations affecting initiation codons may help to understand the potential consequences of these genetic defects, particularly if mutations are identified in patients with a disorder and recombinant models support the clinical data.

The mutation of the initiation methionine codon of *SERPINC1* identified in a thrombophilic family could explain the apparent type I antithrombin deficiency diagnosed in carriers. However, we express this mutation in a eukaryotic cell model aiming to supply further evidences on the consequences of new mutations affecting initiation methionine codons. We also aimed to get additional information that might explain the severe clinical phenotype of carriers (with early and recurrent thrombosis that had fatal consequences in some carriers). Our recombinant studies demonstrate that alternative AUG codons may be used in *SERPINC1* when the initiation methionine is mutated. Two in-frame downstream AUG codons, in a relatively appropriate Kozak context may be used. Interestingly, these alternative downstream AUG codons will generate variant molecules that are expressed at high rates when the wild-type initiation AUG codon is mutated. As these two alternative initiation codons are behind the signal peptide, the proteins generated had no hydrophobic signal peptide and do not enter into the ER. Consequently, these small antithrombins locate in the cytoplasm and they are not glycosylated. Other mutation affecting the signal peptide p.(Leu22Pro) identified in a patient with type I antithrombin deficiency also supports the relevance of the hydrophobic nature of the signal peptide to direct the protein to ER for cotranslational processing events and secretion [19]. We think this mechanism should also affect the other mutation also affecting the initiation AUG codon described in a patient with antithrombin deficiency [10]. There are evidences supporting the use of AUG codons to initiate translation in suboptimal Kozak context [20] and still they are used for initiation without mutations affecting the triplet encoding Met1 [21, 22]. Actually, alternative translation initiation is a mechanism involved in the variability of the human proteome [23, 24]. There are also clues of this bypass mechanism when Met1 is mutated in other proteins [15, 16, 25] but as far as we know this is the first example affecting a serpin. Interestingly, despite of lacking the N-terminal region and N-glycosylation, these small antithrombins are synthesized in a eukaryotic cell line, and have different cellular localization than the native protein. It is required to know the functions that these aberrant antithrombins might play in carriers of this mutation. They do not have anticoagulant function, but it is possible that the reactive center loop of these small antithrombins might be exposed since the protein seems to be cleaved by thrombin. As the N-terminal region of serpins is the portion with minor conservation in the superfamily [26], it is possible that these small antithrombins might be folded, playing a new function. We speculate that the expression of variant antithrombins with different cell location might be involved the severe clinical phenotype of carriers. Patients carrying nonsense mutations in *SERPINC1*, having a high risk of thrombosis, do not usually display the clinical severity shown by this family. Two different mechanisms of gain of function have been suggested to explain the increased clinical severity of mutations associated with antithrombin deficiency, both requiring from generation of an aberrant antithrombin with dominant negative effect that impair the function of the wild-type molecule in heterozygous patients. 1) The deletion of the reactive P1 residue (Arg425) does not only cause a loss of function. This variant (Antithrombin London) has no anticoagulant activity, but also acquires a gain of function: its increased heparin affinity reduces the activation of the wild-type antithrombin by competing to bind heparin. Thus, heterozygous carriers of this mutation have reduced anticoagulant capacity under restricted heparin concentrations. Accordingly, carriers of this mutation have severe thrombotic phenotype that includes early, arterial or recurrent thrombosis [27]. 2) Mutations inducing the folding of antithrombin into hyperstable conformations (latent or polymers) might also reduce the anticoagulant capacity by impairing the function of wild-type molecules. The latent antithrombin forms dimers with native antithrombin [28] and mutations inducing the formation of antithrombin polymers incorporate wild-type monomers into the growing polymers [8]. We speculated that the cytoplasmic aberrant antithrombins generated by the p.Met1? mutation might get a new gain of function that could contribute to the severe clinical phenotype of carriers. Further experiments are required to define the biological relevance of the small antithrombins and to verify such attractive hypothesis. It would also important to clarify in new experiment why these cytosolic aberrant proteins may reach the conditioned media, although there are alternative pathways to secrete cytosolic proteins [29].

Secondly, our study shows new and fascinating data on the initiation of translation in eukaryotes. It is generally assumed that translation occurs in eukaryotic cells at the canonical AUG-methionine codon. However, in certain cases, initiation can occur at codons differing from AUG by a single nucleotide [12]. Our recombinant model in eukaryotic cells revealed that potentially all codons may be used to initiate translation if they are in a proper Kozak context. Additional studies are required to quantify the efficiency of translation of each triplet. Only triplets encoding stop codons are not able to initiate translation. This last result argues against the use of the Met-tRNA to initiate translation independently of the codon present in the RNA [30]. Actually, a recent study suggests that initiation of translation may occur with amino acids encoded by the mutated initiation codon [31]. Unfortunately, the low amount of protein generated in our model, and the processing of the protein, which releases the signal peptide, did not allow identifying the initiation amino acid of the wild-type antithrombin observed in cells transfected with the mutated plasmid. A very recent study demonstrated that other codons than AUG might be used to start the protein synthesis rather than internal methionines in translation-initiation mutants of the ABO blood group [32]. Additionally, the characterization of open reading frames in mouse embryonic stem cells have shown that a great number of alternate initiation sites are non-AUG [33].

In summary, nature is always an excellent source of biological information. The study of a severe thrombophilic family with antithrombin deficiency has identified not only a new mutation in *SERPINC1*, but also new clues on potential new pathological functions of unexpected aberrant proteins and fascinating news on the initiation of translation. The proposed consequences available in mutation databases of mutations affecting the initiation methionine may be incorrect. Two bypassing mechanisms may generate aberrant proteins when mutations affect the initiation methionine: i) downstream methionine codons may be used, resulting in smaller variants (with different post-translational modifications) that might reach abnormal cell localization, like our case; or generate completely different proteins, all with unknown functions and potential unpredictable pathogenic consequences; ii) non-AUG may be used as initiation codons, which may generate proteins with milder (if any) variations. Thus, our study encourages to deep evaluating individually the mutations affecting initiation methionines in different genes, which might have consequences ranging from a mild to a severe effect.

## MATERIALS AND METHODS

### Patients and blood sampling

Blood was collected from the antecubital vein into citrate-tubes and processed within 24 hours after extraction. Plasma was obtained by centrifugation at 2200 g for 20 minutes alicuoted and stored at -80°C. Genomic DNA was purified from mononuclear cells by salting out procedure and stored at -20°C.

The study was approved by the Ethics Committee for Clinical Investigations of the Reina Sofía University Hospital in Murcia (8/2013). All included subjects gave their informed consent to enter the study performed according to the declaration of Helsinki, as amended in Edinburgh in 2000.

### Genetic studies

PCR amplification and sequencing of the seven exons and flanking regions of *SERPINC1* gene were performed essentially as reported [34], from genomic DNA purified from the proband and four relatives with antithrombin deficiency. Other prothrombotic genetic variations (Factor V Leiden, Prothrombin G20210A, and JAK-2 V617F) were also evaluated.

### Measurement of plasma antithrombin activity and antigen levels

Anti-FXa activity of antithrombin was determined by a chromogenic method in citrated plasma. This assay was performed with heparin, bovine FXa, and S-2765 chromogenic substrate (HemosIL TH, Instrumentation Laboratory, Milan, Italy).

Antigen levels were measured by immunodiffusion and a home-made ELISA [35, 36].

Values were express as percentage of those observed in a reference pool of 100 healthy blood donors.

### Electrophoretic characterization of antithrombin

Polyacrylamide gel electrophoresis (PAGE) in denaturing (under reducing and non-reducing conditions) and non-denaturing conditions (both in the presence and absence of 6 M urea) of plasma or recombinant proteins was performed essentially as indicated elsewhere [37]. After separation, proteins were transblotted onto a polyvinylidene difluoride membrane. Antithrombin was immunostained with rabbit anti-human antithrombin polyclonal antibody (Sigma-Aldrich, Madrid, Spain), followed by donkey anti-rabbit IgG–horseradish peroxidase conjugate (GE Healthcare, Barcelona, Spain), with detection via an ECL kit (Amersham Biosciences, Piscataway, NJ, USA).

### Recombinant expression of antithrombin variants

Recombinant antithrombin was constructed on the β-glycoform p.Ser169Ala antithrombin background in order to reduce glycosylation heterogeneity and to facilitate purification.

Site-directed mutagenesis of the pCEP4-S169A antithrombin plasmid, generously donated by Prof. J Huntington, was performed using the Stratagene Quik Change Site-Directed Mutagenesis kit (Agilent Technologies, Santa Clara, CA, USA) as described previously^11^ to generate the p.(Met1Ile) mutation (pCEP4-S169A-M1I) with M1F: GGGTACCGCCACCATTTATTCCAATGTGATAG and M1R: CTATCACATTGGAATAAATGGTGGCGGTACCC primers.

A second wave of site directed mutagenesis was done in the mutated plasmid pCEP4-S169A-M1I. Primers used for site directed mutagenesis are shown in Supplementary Table 1.

Human Embryonic Kidney cells expressing the Epstein Barr Nuclear Antigen 1 (HEK-EBNA) were grown in DMEM with GlutaMAX-I medium (Invitrogen, Barcelona, Spain) supplemented with 5% fetal bovine serum (Sigma-Aldrich, Madrid, Spain) to 60% confluence at 37°C and 5% CO_2_ in a humidified incubator. Transfection was performed by addition 200 μg/mL of plasmid with a previous incubation for 30 min in serum-free OptiMEM culture medium with Lipofectamine LTX reagent (Invitrogen, Madrid, Spain) according to the manufacturer’s protocol. After 24 hours, cells were washed with PBS and exchanged into CD-CHO medium (Invitrogen, Madrid, Spain) supplemented with 4 mM L-glutamine and 0.25 mg/mL Geneticin (Invitrogen, Madrid, Spain). Cells were grown for 10 days and culture medium was collected every 2 days for purification. For all experiments, which were done in triplicate for each mutant, a wild-type plasmid was always used as a reference and positive control using the same conditions. Negative controls, transfection of the same amount of HEK-EBNA cells with vehicle, were always used, although as HEK-EBNA cells do not express antithrombin, transfection of this cell line with vehicle resulted in no expression of antithrombin (data not shown).

The expression of β actin in cell lysates, tested by western blot, was used as gel loading control.

### Recombinant cell protein analyses

Cells were extensively washed with sterile PBS and then lysated with 50 μl of lysis buffer (10 mM TrisHCl, 0.5 mM DTT, 0.035% SDS, 1 mM EGTA, 50 mM sodium fluoride, 50 μM sodium orthovanadate, 5 mM benzamidine and 20 mM phenylmethylsulphonyl fluoride).

The conditioned media and cell lysate was evaluated by SDS-PAGE and Western blot, as indicated before.

Functional activity of antithrombin secreted to the conditioned media was evaluated by incubation with human thrombin (0.25 U) (Calbiochem, Madrid, Spain) and unfractionated heparin (0.6 U) (Rovi, Madrid, Spain) for 30 min at 37°C. Formation of covalent thrombin-antithrombin complex and cleaved antithrombin were detected by Western blotting after denaturing PAGE under reducing or non-reducing conditions, respectively, as previously reported [38].

### Glycomic analysis

Basic glycomic analysis of recombinant proteins from conditioned media was done performed by treatment with Peptide-N-Glycosidase F α(2→3,6,8,9) (PNGase-F) (Roche Diagnostics GmbH, Mannheim, Germany) as previously described[38].

### Protein purification

Purification of recombinant proteins secreted to the conditioned media was performed by heparin chromatography followed by ion-exchange chromatography, as described elsewhere [38].

### Mass spectrometric analyses

Purified recombinant antithrombin was precipitated with 20% TCA on ice and after centrifugation the pellet was washed with cold acetone. Precipitated proteins were resuspended in 25 mM ammonium bicarbonate and digested with 12.5 ng/μl trypsin for 12 hours at 37°C. MS/MS analysis was performed as previously described [39]. Microcapillary reversed phase LC was performed with a CapLCTM (Waters) capillary system. Reversed phase separation of tryptic digests was performed with an Atlantis, C18, 3 μm, 75 μm x 10 cm Nano EaseTM fused silica capillary column (Waters) equilibrated with 5% acetonitrile, 0.2% formic acid. After injection of 6 μl of sample, the column was washed during 5 min with the same buffer and the peptides were eluted using a linear gradient of 5-50% acetonitrile in 120 min at a constant flow rate of 0.2 μl/min. Data processing was performed with MassLynx 4.1. Database searching was done with ProteinLynx Global Server 2.3 (Waters) and Phenyx 2.5 (GeneBio, Geneva, Switzerland) against Uniprot knowledgebase Release 12.3 consisting of UniprotKB/Swiss-Prot Release 54.3 and UniprotKB/TrEMBL Release 37.3.

### Immunofluorescence and immunoelectron microscopy

The intracellular content and distribution of antithrombin by evaluated by immunofluorescence, basically as previously described [40]. Briefly, cells were extensively washed with sterile PBS and then lysated with 50 ml of lysis buffer (10 mM TrisHCl, 0.5 mM DTT, 0.035% SDS, 1 mM EGTA, 50 mM sodium fluoride, 50 mM sodium orthovanadate, 5 mM benzamidine and 20 mM phenylmethylsulphonyl fluoride) and stored at -70°C, prior to analysis. For immunofluorescence analysis, cells were fixed with an equal volume of 4% paraformaldehyde in PBS buffer pH 7.4 (22°C, 20 min). After fixation, cells were washed with PBS, permeabilized with 0.1% Saponin, 0.2% Gelatin, 0.02% Azide (3x5 min). All subsequent incubations and washes contained 0.1% Saponin, 0.2% Gelatin, 0.02% Azide in PBS buffer. Anti-antithrombin antibody (Sigma-Aldrich, Madrid, Spain) was used at 1:1,000 and incubated for 1h at 22°C. Indirect immunofluorescence was carried out using the appropriate fluorescein conjugated goat anti-Rabbit IgG (Vector laboratories, Burlingame, CA, USA) 1:1,000. Fluorescence was analyzed on a LEICA TCS-SP2 microscope using its associated software (Leica Microsystems, Barcelona, Spain).

Cryoimmunoelectron microscopy was performed as described previously [41]. HEK-EBNA cells expressing wild-type or mutant antithrombin were fixed with 2% paraformaldehyde and 0.2% glutaraldehyde in 0.1 M sodium phosphate buffer, pH 7.4. After washing in buffer, the cells were pelleted by centrifugation, embedded in 10% gelatin, cooled on ice and cut into 1 mm^3^ blocks. The blocks were infused with 2.3 M sucrose at 4°C overnight, frozen in liquid nitrogen and stored until cryoultramicrotomy. Sections (∼50 nm-thick) were cut at -120°C with a diamond knife in a Leica Ultracut T/FCS. Ultrathin sections were picked up in a mix of 1.8% methylcellulose and 2.3 M sucrose (1:1). Cryosections were collected on carbon and formvar-coated copper grids and incubated with rabbit polyclonal antibodies against human antithrombin (1:100) followed by protein A-gold. After labelling, the sections were treated with 1% glutaraldehyde, counterstained with uranyl acetate pH 7 and embedded in methyl cellulose-uranyl acetate pH 4 (9:1). Grids were examined with a Jeol JEM-1011 electron microscope.

### Prediction analysis of potential Kozak sequences surrounding potential translation initiation codons

The score of the sequences surrounding potential translation initiation codons to match with the ideal Kozak sequence was evaluated using the TIS Miner Prediction Server software [11].

## SUPPLEMENTARY MATERIALS FIGURES AND TABLE


