# Supplementary material for: Biochemical and cellular consequences of the antithrombin p.Met1? mutation identified in a severe thrombophilic family
Source: Oncotarget. 2018 Sep 4;9(69):33202–14. doi: 10.18632/oncotarget.26059 (PMC6145704; doi:10.18632/oncotarget.26059)
Supplement: Supplementary file 1 [file oncotarget-09-33202-s001.pdf]

Biochemical and cellular consequences of the antithrombin p.Met1? mutation identified in a severe thrombophilic family

SUPPLEMENTARY MATERIALS

Supplementary Table 1: Primers used for site-directed mutagenesis of pCEP4-S137A-M1I plasmid

Code	Sequence	Position	Mut
M5'F	GGGACTTTCCAAAATTTCGTAATAACCCCGC	-40	M-40I
M5'R	GCGGGGTTATTACGAAATTTTGGAAAGTCCC	-40	
R161X-F	GCCAAACTGAACTGCTGACTCTATCGAAAAG	161	R161X
R161X-R	CTTTTCGATAGAGTCAGCAGTTCAGTTTGGC	161	
A0-F	GATCTCTAGAAGGTGGGTACCGCCAC	-5	L-5V
A0-R	GTGGCGGTACCCACCTTCTAGAGATC	-5	
A1-F	GAACCGTCAGATCTCTAGAAGTAGGGTACCGCCACCATTTATTC	-5	L-5X
A1-R	GAATAAATGGTGGCGGTACCCTACTTCTAGAGATCTGACGGTTC	-5	
A2-F	GCTGGGTACCGCCACCTAATATTCCAATGTGATAGG	1	I1X (TAA)
A2-R	CCTATCACATTGGAATATTAGGTGGCGGTACCCAGC	1	
A3-F	GGGTACCGCCACCAGTTATTCCAATGTG	1	I1S
A3-R	CACATTGGAATAACTGGTGGCGGTACCC	1	
A4-F	GTACCGCCACCATTTATTCCTAAGTGATAGGAACTGTAACCTC	4	N4X
A4-R	GAGGTTACAGTTCCTATCACTTAGGAATAAATGGTGGCGGTAC	4	
B2-F	CAGATCTCTAGAAGCTGTGAACCGCCACCATTTATTCC	-4	G-4X
B2-R	GGAATAAATGGTGGCGGTTCACAGCTTCTAGAGATCTG	-4	
B3-F	CTAGAAGCTGGGTACCGCCTGAATTTATTCCAATGTGATAG	-1	T-1X
B3-R	CTATCACATTGGAATAAATTCAGGCGGTACCCAGCTTCTAG	-1	
B4-F	GCTGGGTACCGCCACCGCTTATTCCAATGTGATAG	1	I1A
B4-R	CTATCACATTGGAATAAGCGGTGGCGGTACCCAGC	1	
B6-F	CGCGGGACATTCCCTAGAATCCCATGTGC	49	M49X
B6-R	GCACATGGGATTCTAGGGAATGTCCCGCG	49	
B7-F	CATTCCCATGAATCCCTAGTGCATTTACCGCTCC	52	M52X
B7-R	GGAGCGGTAAATGCACTAGGGATTCATGGGAATG	52	
C1-F	GCTGGGTACCGCCACCTAGTATTCCAATGTGATAGG	1	I1X (TAG)
C1-R	CCTATCACATTGGAATACTAGGTGGCGGTACCCAGC	1	
C2-F	GCTGGGTACCGCCACCTGATATTCCAATGTGATAGG	1	I1X (TGA)
C2-R	CCTATCACATTGGAATATCAGGTGGCGGTACCCAGC	1	
A4-F	GCTGGGTACCGCCACCTGCTATTCCAATGTGATAG	1	I1C
A4-R	CTATCACATTGGAATAGCAGGTGGCGGTACCCAGC	1	
A5-F	GCTGGGTACCGCCACCTACTATTCCAATGTGATAGG	1	I1Y
A5-R	CCTATCACATTGGAATAGTAGGTGGCGGTACCCAGC	1	
A6-F	GGGTACCGCCACCATCTATTCCAATGTGATAG	1	I1I
A6-R	CTATCACATTGGAATAGATGGTGGCGGTACCC	1	
A7-F	CTGGGTACCGCCACCAGGTATTCCAATGTGATAG	1	I1R
A7-R	CTATCACATTGGAATACCTGGTGGCGGTACCCAG	1	
A8-F	CTGGGTACCGCCACCAAGTATTCCAATGTGATAG	1	I1K
A8-R	CTATCACATTGGAATACTTGGTGGCGGTACCCAG	1	

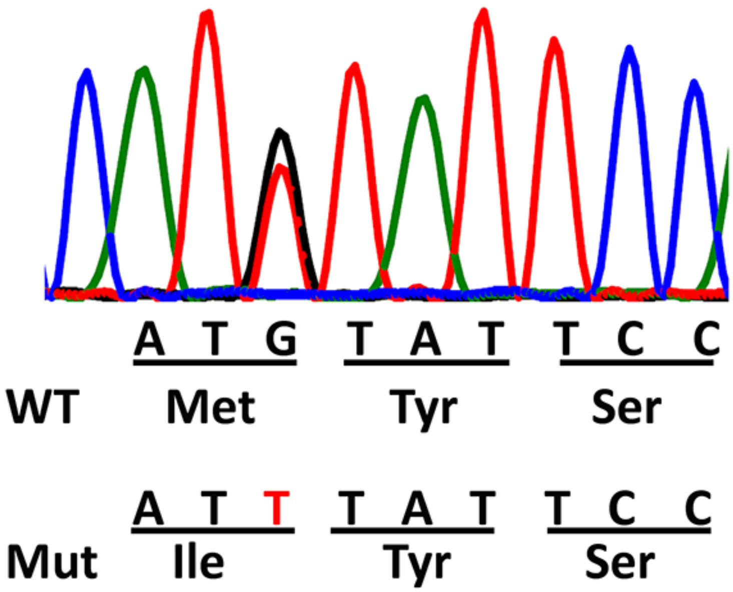

Supplementary Figure 1: Electropherogram of the c.3 G>T heterozygous mutation identified in exon 1 of *SERPINC1* in the proband.

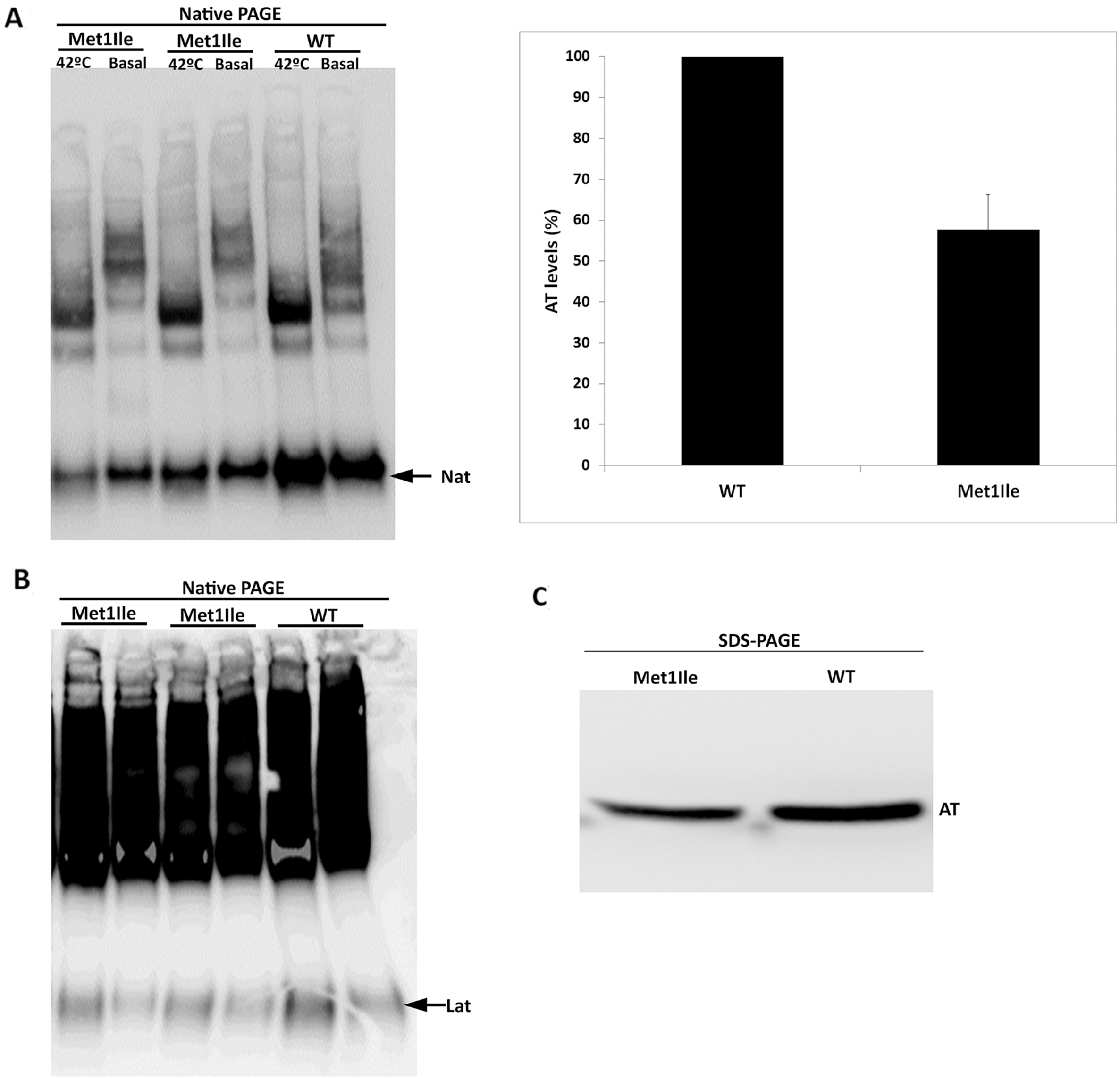

Supplementary Figure 2: Plasma antithrombin of carriers of the p.(Met1Ile) mutation and a pool of 100 healthy controls (WT) detected by Western blot using an anti-human antithrombin polyclonal antibody. (A) Electrophoresis under native conditions with basal samples and after incubation at 42°C for 24 hours (42°C). Native antithrombin (Nat) is pointed by an arrow. Densitometric analysis of basal native antithrombin, done with the Image J software, is also shown. **(B)** Electrophoresis under native conditions with 6M urea, which allows the detection of latent antithrombin (Lat). **(C)** Electrophoresis under denaturing conditions (AT: Antithrombin).

Mascot Search Results

Protein View

```
Match to: ANT3_HUMAN Score: 3543
Antithrombin-III OS=Homo sapiens GN=SERPINC1 PE=1 SV=1
Found in search of D:\interc\PKLM_2012\JavierCorral_23102013_1.pkl

Nominal mass (Mr): 53025; Calculated pI value: 6.32
NCBI BLAST search of ANT3_HUMAN against nr
Unformatted sequence string for pasting into other applications

Fixed modifications: Carbamidomethyl (C)
Variable modifications: Oxidation (M)
Cleavage by Trypsin: cuts C-term side of KR unless next residue is P
Sequence Coverage: 57%

Matched peptides shown in Bold Red

  1 MYSNVIGTVT SGKRKVYLLS LLLIGFWDCV TCHGSPVDIC TAKPRDIPMN
 51 PMCIYRSPEK KATEDEGSEQ KIPEATNRRV WELSKANSRF ATTFYQHLAD
101 SKNDNDNIFL SPLSISTAFA MTKLGACNDT LQQLMEVFKF DTISEKTSDQ
151 IHFFFAKLNC RLYRKANKSS KLVSANRLFG DKSLTFNETY QDISELVYGA
201 KLQPLDFKEN AEQSRAAINK WVSNKTEGRI TDVIPSEAIN ELTVLVLVNT
251 IYFKGLWKSK FSPENTRKEL FYKADGESCS ASMMYQEGKF RYRRVAEGTQ
301 VLELPFKGDD ITMVLILPKP EKSLAKVEKE LTPEVLQEWL DELEEMMLVV
351 HMPRFRIEDG FSLKEQLQDM GLVDLFSPEK SKLPGIVAEG RDDLYVSDAF
401 HKAFLEVNEE GSEAAASTAV VIAGRSLNPN RVTFKANRPF LVFIREVPLN
451 TIIFMGRVAN PCVK
```

Supplementary Figure 3: Mascot search results from the proteomic analysis of the small recombinant antithrombins purified from conditioned media of cells transfected with the mutant plasmid. Peptides of antithrombin identified by this analysis are marked in red.

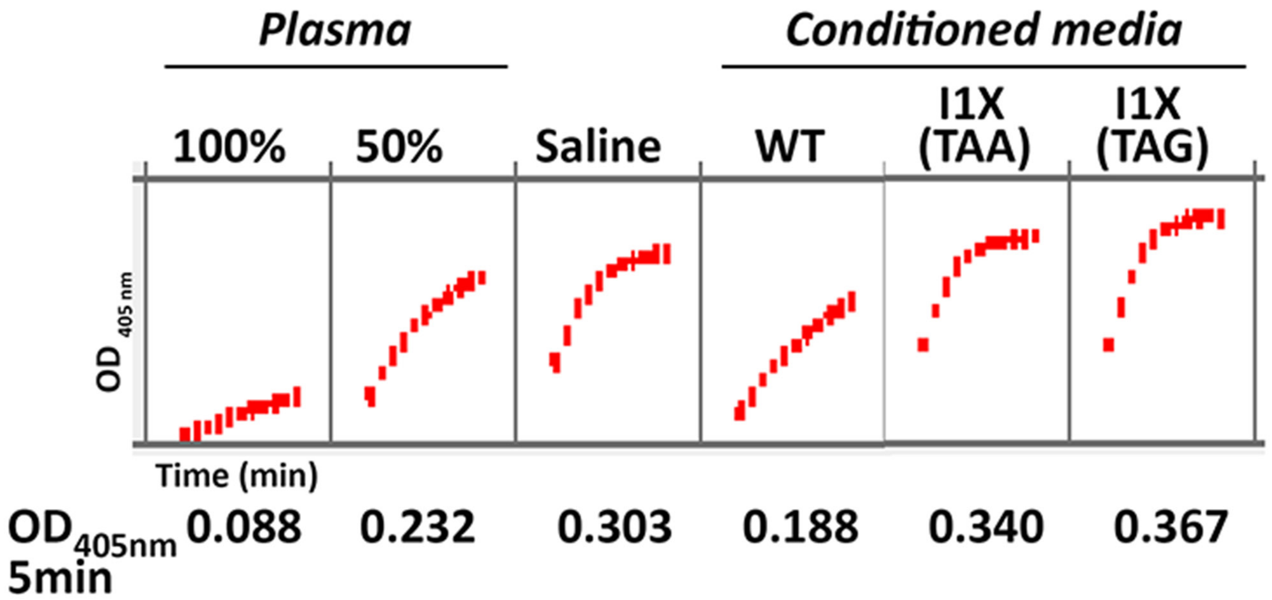

Supplementary Figure 4: Anti-FXa activity of recombinant antithrombins from conditioned medium of HEK-EBNA cells transfected with pCEP4-S169A (wild type WT antithrombin), pCEP4-S169A-I1X (TAA) and pCEP4-S169A-I1X (TAG) plasmids generating only small antithrombins. 30x of conditioned media was used for mutant antithrombins. As controls, plasma from a healthy subject and a negative control (saline) were used. A chromogenic method was used to determine anti-FXa activity. Absorbance at 405nm is shown. The OD values observed at 5 minutes are also indicated.

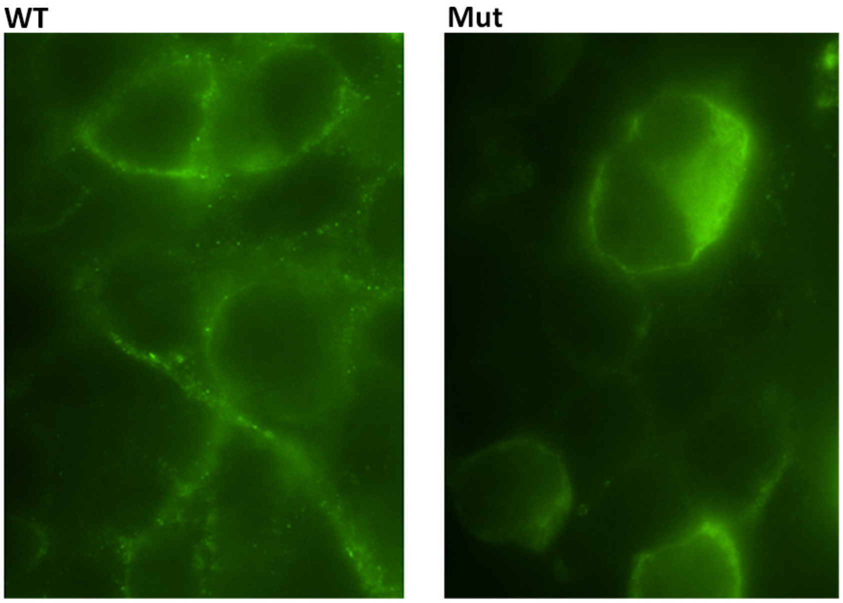

Supplementary Figure 5: Immunofluorescence analysis of antithrombin expression of HEK-EBNA cells 24 h after transfection with pCEP4-S169A (wild type WT antithrombin) and pCEP4-S169A-M1I (Mut) plasmids.
